# Mapping of hormones and cortisol responses in patients after Lyme neuroborreliosis

**DOI:** 10.1186/1471-2334-10-20

**Published:** 2010-02-05

**Authors:** Ivar Tjernberg, Martin Carlsson, Jan Ernerudh, Ingvar Eliasson, Pia Forsberg

**Affiliations:** 1 Department of Clinical Chemistry, Kalmar County Hospital, SE-391 85 Kalmar, Sweden; 2 Division of Infectious Diseases, Department of Clinical and Experimental Medicine, Linköping University, SE-581 83 Linköping, Sweden; 3 Division of Clinical Immunology, Unit of Autoimmunity and Immune Regulation (AIR), Department of Clinical and Experimental Medicine, Linköping University, SE-581 83 Linköping, Sweden; 4 Department of Laboratory Medicine, NÄL, SE-461 85 Trollhättan, Sweden

## Abstract

**Background:**

Persistent symptoms after treatment for neuroborreliosis are common for reasons mainly unknown. These symptoms are often unspecific and could be caused by dysfunctions in endocrine systems, an issue that has not been previously addressed systematically. We therefore mapped hormone levels in patients with previous confirmed Lyme neuroborreliosis of different outcomes and compared them with a healthy control group.

**Methods:**

Twenty patients of a retrospective cohort of patients treated for definite Lyme neuroborreliosis were recruited 2.3 to 3.7 years (median 2.7) after diagnosis, together with 23 healthy controls. Lyme neuroborreliosis patients were stratified into two groups according to a symptom/sign score. All participants underwent anthropometric and physiological investigation as well as an extensive biochemical endocrine investigation including a short high-dose adrenocorticotropic hormone stimulation (Synacthen^®^) test. In addition to hormonal status, we also examined electrolytes, 25-hydroxy-vitamin D and interleukin-6.

**Results:**

Eight patients (40%) had pronounced symptoms 2-3 years after treatment. This group had a higher cortisol response to synacthen as compared with both controls and the Lyme neuroborreliosis patients without remaining symptoms (p < 0.001 for both comparisons). No other significant differences in the various baseline biochemical parameters, anthropometric or physiological data could be detected across groups.

**Conclusions:**

Apart from a positive association between the occurrence of long-lasting complaints after Lyme neuroborreliosis and cortisol response to synacthen, no corticotropic insufficiency or other serious hormonal dysfunction was found to be associated with remaining symptoms after treatment for Lyme neuroborreliosis.

## Background

Lyme borreliosis is the most commonly reported tick-transmitted disease in the northern hemisphere [1]. The overall incidence in the south of Sweden has been reported to be 69 per 100 000 inhabitants and year, with marked regional variability. For instance, the incidence in Kalmar County was reported to be 160 per 100 000 inhabitants and year [2].

Clinical manifestations of Lyme borreliosis are diverse and include erythema migrans, neuroborreliosis, arthritis, lymphocytoma, carditis, and acrodermatitis chronica atrophicans. In Sweden, Lyme neuroborreliosis (LNB) is the second most common manifestation after erythema migrans. Current antibiotic treatment recommendations for LNB comprise either doxycycline, benzyl penicillin or ceftriaxone [1-3].

Residual symptoms after treatment of LNB are common and have been reported by some 25-50% of patients 6-142 months after onset of neurological symptoms [3-5]. Residual symptoms may include arthralgia, musculoskeletal or radicular pain, paresthesia, dysaesthesia together with persistent fatigue and neurocognitive impairment. Although there are theories regarding the background of these persistent symptoms, the pathogenesis is still mainly unknown [6,7]. Early recognition and treatment of acute LNB seems to be important in order to reduce the risk of persistent symptoms [3].

It is well known that tuberculosis meningitis and various other infectious diseases can affect and cause dysfunction of the hypothalamus and/or the pituitary gland [8-16]. It has also been proposed that interleukin-6 (IL-6) may stimulate the hypothalamus-pituitary-adrenal (HPA) axis during inflammatory stress [17], and in healthy individuals a positive correlation between IL-6 levels and cortisol response after a standard short low-dose (1 μg) adrenocorticotropic hormone (ACTH) test has been reported [18].

In a recent study, isolated corticotropic insufficiency was noted in four of 19 patients (21%) with previous infectious diseases of the central nervous system. The study in fact included four patients with a history of LNB, but no hormonal dysfunction was found in these [19]. Endocrine dysfunctions, with general symptoms and complaints could be one possible explanation, and vitamin D deficiency, linked to diffuse musculoskeletal complaints, could be another explanation for the reported complaints after LNB [20]. Neither of these has been thoroughly investigated in this context previously.

In the present study we therefore aimed at mapping patients having experienced confirmed LNB with and without persistent symptoms and to compare them with healthy controls regarding anthropometric data, various hormone levels, and, in particular, the HPA axis. In addition, we wanted to relate the high-dose ACTH stimulation (Synacthen^®^) test-induced cortisol response to the level of symptoms as well as to levels of IL-6.

## Methods

### Patients and controls

From the routine laboratory data system of the Department of Microbiology at Kalmar County Hospital, 36 adult patients were identified as having had a positive anti-borrelia antibody index (AI; IDEA™ Lyme Neuroborreliosis kit, Oxoid, Basingstoke, United Kingdom) with purified, native *Borrelia afzelii *strain DK1 flagellum as test antigen in paired cerebrospinal fluid/serum samples during 2004-2005. According to medical charts at Kalmar County Hospital, seven of these 36 patients were not considered suffering from ongoing LNB. These seven patients were all considered to have other diagnoses based on clinical history, symptoms and laboratory findings.

The remaining 29 patients received a clinical diagnosis of LNB and were treated with doxycycline, in one case together with ceftriaxone. Painful radiculitis was found in 19, unspecific complaints in 11, cranial nerve palsy in 9 and meningitis in 2 patients. Each patient presented with one or multiple features. An IgM or IgG AI of ≥ 0.3 was considered positive according to the manufacturer. Positive IgM AI was found in 13/29 patients (range 0-20.5; median 0.1), and positive IgG AI was found in 29/29 patients (range 0.4-973.4; median 15.8). Positive blood serological results were found in 26 of the 29 patients (Immunetics^® ^Quick ELISA C6 Borrelia Assay Kit, Immunetics Inc., Cambridge, MA, USA).

Although six of these 29 patients lacked pleocytosis (>5*10^6 ^leukocytes/L) of the cerebrospinal fluid, they were still clinically diagnosed with LNB according to medical records, based on a typical history of symptoms and findings together with the positive AI, and were therefore treated.

Individuals in this retrospective cohort, consisting of 29 patients, were informed and offered participation in the study. Twenty of these 29 (69%) patients participated in the study during February-May 2008. One additional patient accepted participation, but was on medication with prednisolone due to suspected polymyalgia rheumatica and was therefore not included in the study. The remaining eight patients were reminded by letter after a couple of weeks, but no additional participants were recruited.

In addition, a control group of healthy adult blood donors was recruited. Only subjects with an approved declaration of health and no history of previous Lyme borreliosis infection and a negative borrelia serological test (C6 Lyme ELISA™ Kit, Immunetics^®^, Boston, MA, USA) were included. Controls using medications, working night shift or having had any previous hormonal disease were also not included. Finally, 23 healthy control individuals participated in the study during February-May 2008. The study was performed according to the Declaration of Helsinki, and the study protocol was reviewed and approved by the regional ethical review board in Linköping, Sweden. All patients and controls gave informed consent.

### Study protocol and implementation

A study protocol was designed for the LNB patients in whom 15 different neurological signs together with 25 different symptoms were addressed. Blood pressure, heart rate, weight, length and waist measurement were noted and fasting (minimum 8 hours) blood samples were collected between 8 and 9 a.m. from all participants.

Then, an intramuscular injection with 0.25 mg of Synacthen^® ^(Novartis Pharma AG Basel, Switzerland) was given after which samples for cortisol were taken at 30 and 60 minutes. An experienced nurse, with access to a physician, administered synacthen and supervised the participants. No adverse events occurred during the test. Directly after the injection all LNB patients were neurologically examined and questioned by the same physician (IT) regarding persistent, ongoing symptoms after treatment of LNB. Only neurological signs and symptoms related by the patient to the LNB infection were noted. The neurological examination included systematic testing of cranial nerves, motor and sensory functions, balance, ataxia and reflexes. All results were noted in the study protocol, and all participants were unaware of the other study results. Analyses were performed according to methods and instruments in Additional file 1, Table S1, where reference values also are noted. The highest of the 30 and 60 minute values of synacthen stimulated cortisol minus baseline cortisol level was defined as the absolute cortisol increment (ACI).

### Statistical analysis

Differences between groups were analysed with Kruskal-Wallis non-parametrical test, followed by Mann-Whitney's U-test in case of significance. Due to age differences between blood donors (controls) and LNB patients, adjusting for age was made using factorial ANOVA for the normally distributed parameters (ACI, heart rate and systolic blood pressure) followed by Duncan's test in case of significance. Pearson correlation was used to analyse the coupling between post treatment severity (see results) score and number of symptoms on the one hand and ACI as well as IL-6 values (log-normally distributed, thus using the logarithm values) on the other. A significance level of p < 0.05 was considered statistically significant. Statistica 8.0 was used for statistical calculations.

## Results

### Symptoms and neurological signs

Twenty LNB patients were included in the study and the median follow-up time from diagnosis was 2.7 years (range 2.3 to 3.7). The most common long-lasting symptoms were arthralgia (40%), balance disorder (35%) and paresthesia (35%) (Additional file 2, Table S2). In the neurological examination, three patients were not fully recovered after facial palsy. No other pathological neurological signs were noted in these patients. In four patients, ataxia, disturbed motor and sensory nerve functions were found (data not shown).

By adding the numbers of symptoms (range 0-25; in the questionnaire) to the number of pathological signs in the neurological examination (range 0-15; according to the study protocol), a post treatment severity (PTS) score was calculated for each LNB patient (range 0-40).

**Figure 1:**
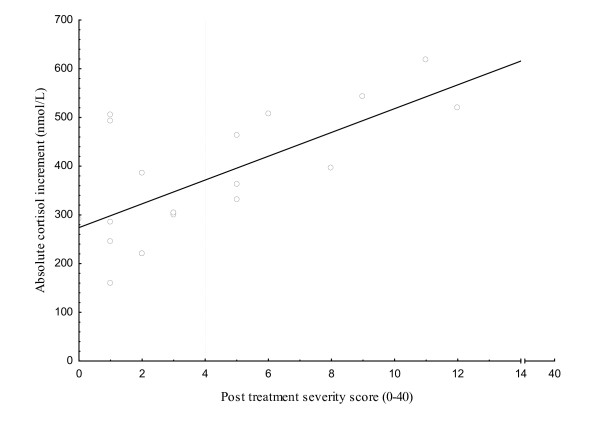
**Symptoms and neurological signs compared to absolute cortisol increment in Lyme neuroborreliosis patients post treatment**. Numbers of symptoms and neurological signs at follow up 2.3 to 3.7 years post treatment (x-axis) and absolute cortisol increment (ACI) after 0.25 mg Synacthen^® ^injection (y-axis) in Lyme neuroborreliosis patients (n = 20). Pearson correlation coefficient r = 0.68; p = 0.001. The post treatment severity (PTS) score was calculated by adding the number of symptoms and pathological neurological signs strictly related to the Lyme neuroborreliosis infection for each patient. The dotted line at score = 4 represents a chosen cut off. Patients with a score above 4 were considered having more pronounced complaints (LNB^+ ^n = 8) and below four not pronounced complaints (LNB^- ^n = 12).

Based on the PTS score, patients could be divided into two groups with more or less pronounced symptoms after LNB. An arbitrarily chosen cut off level of four discriminated the patients in two clusters (Figure 1) and gave the most significant difference regarding ACI across groups: Patients having a score less than four (LNB^-^, n = 12) and patients with a score above four (LNB^+^, n = 8). Based on this system, 8/20 (40%) of the LNB patients were classified as having more pronounced long-lasting symptoms in spite of treatment for LNB according to recommendations [3]. The distribution of symptoms in the LNB subgroups is also shown in Additional file 2, Table S2.

### Physiological and biochemical results

Anthropometric and physiological parameters such as body measurements, heart rate and blood pressure is shown together with biochemical results in controls and in the LNB^+ ^and LNB^- ^groups in Additional file 3, Table S3. In a first step, comparisons between all three groups were performed using Kruskal-Wallis test, showing that age (p = 0.002), heart rate (p = 0.010), systolic blood pressure (p = 0.011), follicle-stimulating hormone (FSH; p = 0.033), ACI (p = 0.004) and IL-6 (p = 0.048) were statistically different across groups. Further analyses using Mann-Whitney's U-test showed that controls were younger than LNB^+ ^and LNB^- ^patients (p = 0.001 and 0.023, respectively), but no difference in age was noted in between LNB^+ ^and LNB^- ^patients. Also, higher ACI was found in the LNB^+ ^group compared to both other groups (p = 0.002 for both comparisons), while no differences were found regarding basal concentrations of cortisol or metabolic parameters such as waist measurement or fasting glucose. Heart rate was also found to be higher in the LNB^+ ^group compared to both controls and LNB^- ^groups (p = 0.004 and 0.006, respectively). Furthermore, higher systolic blood pressure (p = 0.003) and levels of FSH (p = 0.01) were noted in the LNB^+ ^group compared to controls. Concentrations of FSH in women are dependent on time of menstruation and rise after menopause and are therefore difficult to compare among groups. Because of the difference in age between groups, statistical correction for age was done. Thereby, ACI still remained higher in the LNB^+ ^group compared to both controls and the LNB^- ^group (p < 0.001 for both comparisons). However, no differences between groups were detected regarding IL-6, heart rate or systolic blood pressure after age correction. Since a diagnosis of LNB without pleocytosis has been debated, we also, as an extra precaution, reanalysed the coupling between PTS score and ACI as well as differences in ACI between groups after exclusion of 4/20 LNB patients (2 LNB^+^, 2 LNB^-^) with normal CSF cell counts, with similar results.

### Correlations

Correlations between PTS score and ACI (Figure 1) showed that ACI for LNB patients was significantly correlated to the PTS score (r = 0.68; p = 0.001). The chosen PTS score cut off level of four is also shown in Figure 1, separating the LNB patients into the two subgroups. No correlation between IL-6 and PTS score or IL-6 and ACI was found, not even for healthy controls only (data not shown). Finally, we also determined correlations in LNB patients classified according to self reported symptoms only, i.e. excluding signs in the PTS score. Still, a significant correlation between ACI and the added number of symptoms was found (r = 0.65; p = 0.002, data not shown). No correlation between ACI and the number of pathological neurological signs was found.

### Complementary results

All LNB^+ ^and LNB^- ^patients had corticotrophin-releasing hormone (CRH) levels < 1.0 pmol/L, as well as all controls except two (1.2 and 1.9 pmol/L, respectively).

Two of the controls were found to have subclinical autoimmune hypothyroidism based on increased levels of TSH (4.3 and 11.5 mU/L) combined with a decreased level of thyroxine in one case (6.4 pmol/L), as well as positive levels of anti-thyroid peroxidase antibodies in both cases.

One LNB^- ^patient was found to have an increased level of ionized calcium and was after further investigation diagnosed with primary hyperparathyroidism.

## Discussion

We found no significant differences regarding various basal hormonal concentrations when comparing LNB patients of different clinical outcomes 2-3 years post treatment to controls. This finding is clinically relevant in that it indicates that endocrine dysfunction is not a major cause of long-lasting symptoms despite optimal treatment of LNB. However, a positive correlation between the PTS score and ACI was noted. After sub grouping and corrections for age the LNB^+ ^group still had higher ACI than the LNB^- ^group and healthy controls, whereas no differences were found in metabolic parameters such as waist measurement or fasting glucose.

In a recent study it was shown that ACI correlated positively to IL-6 levels in normal subjects [18], and therefore higher levels of IL-6 in LNB patients with more pronounced symptoms could be expected to correlate with higher ACI. Associations between IL-6 and depression, vital exhaustion and feeling of hopelessness have also been previously reported [21-23]. However, in the present study we did not find a significant correlation between PTS score and IL-6 or between ACI and IL-6, not even when studying the control group only, thereby contradicting the results of Zarkovic et al [18]. We therefore interpret the correlation between patient score and ACI in this study as independent of IL-6 levels. It is however interesting to notice the relation between clinical symptoms and cortisol response to synacthen indicating adrenal hyper-responsiveness in these patients. Some earlier studies have shown super-sensitivity to ACTH stimulation with higher cortisol responses in depressed patients compared to controls [24-26], while others have been unable to differentiate between depressed patients and healthy volunteers [27,28]. Cortisol response to ACTH stimulation and adrenal gland volume has also been shown to be state-dependent with decreased cortisol response to ACTH and decreased adrenal volume in successfully treated depressed patients [29,30]. Although a complete evaluation of depression in the LNB patients in this study was not performed, obvious depressive symptoms according to Additonal file 2, Table S2, were not the most common and therefore other causes for the higher ACI in the LNB+ patients may be suspected. It has also been reported that chronic exposure to stressful situations, perhaps such as the persistent symptoms noted in this study, results in tonic changes in the HPA axis [31]. Taken together, further investigation is necessary in order to determine the cause of increased ACI in LNB patients with persistent symptoms and the relation to LNB, depression, stress signalling, cortisol and adrenal gland volume.

In this study, as much as 8/20 (40%) of LNB patients were considered to suffer from persistent symptoms between 2-3 years post treatment. This is in line with previously reported proportions (25-50%) of patients suffering from persistent symptoms after treatment for LNB [3-5]. The figure of 40% could however be overestimated in this study due to an overrepresentation of patients with symptoms participating in the study. It can not be excluded that those eight patients that chose to not participate, were patients with minor or no persistent symptoms and that they therefore were less motivated to participate. Six of those eight patients were younger than 54 years of age, and may also have chosen not to participate due to working conditions as the whole investigation took around two hours of time. One other weakness of this study is that controls are significantly younger than LNB patients. This is because blood donors served as controls, and only few blood donors between 65-70 years of age and no donors above 70 years of age were available for participation. The differences of age between groups were therefore considered and age correction performed in the statistical analyses when appropriate.

## Conclusion

In conclusion, this study showed that a high proportion (40%) of LNB patients suffer from persistent symptoms 2-3 years post treatment. Although a positive correlation between ACI and PTS score was found in the LNB group, no major differences regarding various basal hormone levels could be detected comparing the LNB subgroups and the control group. This finding indicates that endocrine dysfunction is not a major cause for long-lasting post-treatment symptoms in LNB.

## Competing interests

The authors declare that they have no competing interests.

## **Authors' contributions**

IT conceived of the study, participated in its design and coordination and helped draft the manuscript. MC participated in the study design, data analysis and helped draft the manuscript. JE participated in the study design, data analysis and helped draft the manuscript. IE helped draft the manuscript. PF participated in the study design, data analysis and helped draft the manuscript. All authors read and approved the final manuscript.

## Pre-publication history

The pre-publication history for this paper can be accessed here:

http://www.biomedcentral.com/1471-2334/10/20/prepub

## Supplementary Material

Additional file 1 Analyses, reference values, methods and instruments used. Click here for file

Additional file 2 **Symptoms in Lyme neuroborreliosis patients 2-3 years post treatment**. Persistent subjective symptoms in patients related to Lyme neuroborreliosis 2.3 to 3.7 years post treatment according to questionnaire (n = 20). Click here for file

Additional file 3 **Anthropometric, physiological and biochemical parameters in controls and Lyme neuroborreliosis patients 2-3 years post treatment**. Anthropometric, physiological and biochemical parameters in healthy controls (n = 23) and Lyme neuroborreliosis patients without (LNB^- ^n = 12) or with (LNB^+ ^n = 8) pronounced symptoms and neurological signs at follow up 2.3 to 3.7 years post treatment. After correction for age, absolute cortisol increment remained significantly higher in the LNB^+ ^group compared to both controls and the LNB^- ^group (p < 0.001). Click here for file
