# Supplementary material for: Mapping of hormones and cortisol responses in patients after Lyme neuroborreliosis
Source: BMC Infect Dis. 2010 Feb 5;10:20. doi: 10.1186/1471-2334-10-20 (PMC2827415; doi:10.1186/1471-2334-10-20)
Supplement: Additional file 1 — Analyses, reference values, methods and instruments used. [file 1471-2334-10-20-S1.PDF]

Analysis	Reference values and units	Instrument / Principle, Company / Department
P-Glucose	4.2 - 6.1 mmol/L (fasting)	Vitros 5,1 FS Chemistry System, Ortho Clinical Diagnostics
P-Sodium	137 - 145 mmol/L	Vitros 5,1 FS Chemistry System, Ortho Clinical Diagnostics
P-Potassium	3.5 - 4.4 mmol/L	Vitros 5,1 FS Chemistry System, Ortho Clinical Diagnostics
P-Creatinine	60 - 100 (male), 50 - 90 (female) µmol/L	Vitros 5,1 FS Chemistry System, Ortho Clinical Diagnostics
S-Ionized calcium	1.18 - 1.31 mmol/L	ABL 825, Radiometer Medical ApS
S-Follicle-stimulating hormone	1.3 - 19 (male), 1.8 - 23 (female[a]), 17 - 114 (post menopause) IU/L	Access 2, Beckman Coulter
S-Luteinizing hormone	1.2 - 8.6 (male), 1.2 - 103 (female[a]), 11 - 59 (post menopause) IU/L	Access 2, Beckman Coulter
S-Prolactin	2.6 - 13 (male), 3.3 - 27 (female pre menopause), 2.7 - 20 (post menopause) µg/L	Access 2, Beckman Coulter
S-Cortisol	140 - 700 (unstimulated morning values) nmol/L	UniCel DxI, Beckman Coulter
S-free thyroxine	8 - 14 pmol/L	UniCel DxI, Beckman Coulter
S-Thyrotrophin	0.4 - 3.5 mU/L	UniCel DxI, Beckman Coulter
S-25-hydroxy-vitamin D3	> 75 nmol/L	HPLC, Shimadzu Europé
P-Adenocorticotropin	2.0 - 10 pmol/L	Immulite 2000, Siemens Healthcare Diagnostics
S-Insulin-like growth factor I	85 - 420[b] µg/L	Immulite 2000, Siemens Healthcare Diagnostics
P-Corticotrophin releasing hormone	< 5 pmol/L	RIA, Gammacounter, Wallac
S-Interleukin-6	Not available	hsELISA, R&D Systems

P = Plasma
S = Serum
Reference values apply to individuals of 18 years of age or more.
[a] Reference values depend on sampling time in the menstrual cycle.
[b] Reference values depend on age.
HPLC = High performance liquid chromatography
RIA = Radioimmunoassay
hsELISA = high sensitivity Enzyme-Linked ImmunoSorbent Assay
