# Supplementary material for: Mapping of hormones and cortisol responses in patients after Lyme neuroborreliosis
Source: BMC Infect Dis. 2010 Feb 5;10:20. doi: 10.1186/1471-2334-10-20 (PMC2827415; doi:10.1186/1471-2334-10-20)
Supplement: Additional file 2 — Symptoms in Lyme neuroborreliosis patients 2-3 years post treatment. Persistent subjective symptoms in patients related to Lyme neuroborreliosis 2.3 to 3.7 years post treatment according to questionnaire (n = 20). [file 1471-2334-10-20-S2.PDF]

Symptom	LNB all (n=20; 9F, 11M)<br>Number (%)	LNB$^-$ (n=12; 4F, 8M)<br>Number (%)	LNB$^+$ (n=8; 5F, 3M)<br>Number (%)
Arthralgia	8 (40)	2 (17)	6 (75)
Balance disorder	7 (35)	2 (17)	5 (62)
Paresthesia	7 (35)	3 (25)	4 (50)
Myalgia	6 (30)	1 (8)	5 (62)
Decreased libido	6 (30)	1 (8)	5 (62)
Memory problems	5 (25)	2 (17)	3 (38)
Headache	4 (20)	0 (0)	4 (50)
Weakness	3 (15)	0 (0)	3 (38)
Freezing	3 (15)	1 (8)	2 (25)
Attention problems	3 (15)	0 (0)	3 (38)
Painful radiculitis	3 (15)	0 (0)	3 (38)
Facial palsy	3 (15)	3 (25)	0 (0)
Sleeping problems	3 (15)	0 (0)	3 (38)
Unexplained weight-gain	2 (10)	0 (0)	2 (25)
Hearing impairment	2 (10)	1 (8)	1 (12)
Other neuritis	2 (10)	1 (8)	1 (12)
Tremor	2 (10)	0 (0)	2 (25)
Nausea	1 (5)	0 (0)	1 (12)
Depression	1 (5)	0 (0)	1 (12)
Oedema	1 (5)	0 (0)	1 (12)
Unexplained weight-loss	0 (0)	0 (0)	0 (0)
Fever	0 (0)	0 (0)	0 (0)
Motor weakness arm/leg	0 (0)	0 (0)	0 (0)
Decreased appetite	0 (0)	0 (0)	0 (0)
Irregular menstruations	0 (0)	0 (0)	0 (0)

F = female, M = male

LNB$^-$ = Lyme neuroborreliosis with less than four parallel persistent symptoms and/or neurological findings post treatment.

LNB$^+$ = Lyme neuroborreliosis with more than four parallel persistent symptoms and/or neurological findings post treatment.

Only symptoms identified by the patients as strictly related to the Lyme neuroborreliosis infection were registered.
