# Supplementary material for: Mapping of hormones and cortisol responses in patients after Lyme neuroborreliosis
Source: BMC Infect Dis. 2010 Feb 5;10:20. doi: 10.1186/1471-2334-10-20 (PMC2827415; doi:10.1186/1471-2334-10-20)
Supplement: Additional file 3 — Anthropometric, physiological and biochemical parameters in controls and Lyme neuroborreliosis patients 2-3 years post treatment. Anthropometric, physiological and biochemical parameters in healthy controls (n = 23) and Lyme neuroborreliosis patients without (LNB- n = 12) or with (LNB+ n = 8) pronounced symptoms and neurological signs at follow up 2.3 to 3.7 years post treatment. After correction for age, absolute cortisol increment remained significantly higher in the LNB+ group compared to both controls and the LNB- group (p < 0.001). [file 1471-2334-10-20-S3.PDF]

	Healthy controls (n=23 ; 12 females, 11 males)			LNB^{-} (n=12 ; 4 females, 8 males)			LNB^{+} (n=8 ; 5 females, 3 males)			Kruskal-Wallis
	Median	Q1-Q3	Range	Median	Q1-Q3	Range	Median	Q1-Q3	Range	p-value
Age (years)	49	33 - 60	22 - 70	59	48 - 71	34 - 77	66	60 - 72	57 - 85	0.002 *
Length (cm)	170	166 - 180	158 - 189	180	169 - 183	162 - 191	170	166 - 179	158 - 184	0.616
Weight (kg)	74	64 - 101	56 - 120	81	70 - 91	55 - 116	75	66 - 80	58 - 87	0.640
BMI (kg/m2)	25.6	22.8 - 29.1	19.4 - 37.0	26.8	24.3 - 27.8	19.3 - 31.8	24.0	22.6 - 27.8	20.1 - 31.8	0.740
Waist measurement all (cm)	85	79 - 102	64 - 115	93	87 - 97	78 - 106	90	84 - 96	77 - 100	0.544
Waist measurement women (cm)	80	74 - 87	64 - 110	84	81 - 92	78 - 98	85	83 - 96	77 - 100	0.590
Waist measurement men (cm)	93	82 - 104	80 - 115	95	91 - 97	89 - 106	93	86 - 96	86 - 96	0.890
Heart rate (beats/min) [a]	62	60 - 64	48 - 80	64	60 - 64	48 - 80	68	68 - 80	64 - 84	0.010 **
Systolic blood pressure (mm Hg)	120	110 - 140	95 - 160	130	120 - 145	105 - 160	150	130 - 155	120 - 170	0.011 ***
Diastolic blood pressure (mm Hg)	75	70 - 80	60 - 95	78	73 - 80	55 - 90	80	80 - 85	65 - 90	0.135
Glucose (mmol/L)	5.0	4.6 - 5.5	3.7 - 6.1	5.0	4.5 - 5.4	3.7 - 5.5	5.0	4.7 - 5.4	4.3 - 5.7	0.974
Potassium (mmol/L) [b]	3.9	3.7 - 4.0	3.5 - 4.3	3.8	3.7 - 4.2	3.6 - 4.3	3.9	3.7 - 4.4	3.5 - 4.8	0.759
Creatinine (µmol/L) [b]	73	65 - 81	53 - 96	74	70 - 81	44 - 102	71	65 - 84	49 - 106	0.869
Sodium (mmol/L) [b]	141	139 - 143	137 - 145	141	140 - 143	139 - 144	142	141 - 143	141 - 146	0.283
Ionized calcium (mmol/L)	1.25	1.22 - 1.28	1.17 - 1.30	1.27	1.24 - 1.30	1.21 - 1.43	1.26	1.24 - 1.28	1.20 - 1.34	0.466
Follicle stimulating hormone (IU/L)	5.5	3.2 - 14.5	1.6 - 89.8	9.2	4.3 - 54.6	3.5 - 80.8	53.2	13.3 - 70.1	3.6 - 97.7	0.033 ***
Basal cortisol (nmol/L)	410	336 - 459	175 - 563	376	348 - 502	280 - 673	380	291 - 519	228 - 615	0.931
Stimulated cortisol at 30 minutes (nmol/L)	608	571 - 696	487 - 816	661	569 - 760	510 - 850	744	637 - 881	540 - 977	0.099
Stimulated cortisol at 60 minutes (nmol/L)	691	658 - 761	580 - 972	757	566 - 838	530 - 978	860	727 - 1029	624 - 1109	0.089
Absolute cortisol increment (ACI; nmol/L)	312	264 - 381	149 - 494	296	233 - 346	160 - 506	485	379 - 532	332 - 618	0.004 **
Luteinizing hormone (IU/L)	5	2 - 6	1 - 36	4	3 - 22	2 - 44	21	3 - 27	2 - 29	0.282
Prolactin (µg/L)	7.9	5.6 - 12.2	3.4 - 23.2	7.8	5.9 - 10.6	4.6 - 37.4	8.2	4.7 - 9.9	3.6 - 14.7	0.806
Free thyroxine (pmol/L)	9.6	8.8 - 10.5	6.5 - 12.9	10.7	9.7 - 11.3	9.0 - 12.4	10.7	9.0 - 11.2	8.1 - 12.8	0.183
Thyrotrophin (mU/L)	2.4	1.6 - 3.2	0.3 - 11.5	2.1	1.5 - 2.7	1.1 - 4.5	1.9	1.3 - 2.3	0.9 - 3.0	0.539
Adrenocorticotropin (pmol/L)	5.1	3.7 - 5.7	2.0 - 9.3	4.6	3.6 - 6.2	3.0 - 8.0	3.8	2.8 - 4.5	1.8 - 6.0	0.136
Insulin-like growth factor-I (µg/L)	170	120 - 213	66 - 260	108	94 - 125	66 - 196	101	86 - 197	75 - 228	0.078
25-hydroxy vitamin D3 (nmol/L)	83	55 - 97	39 - 151	84	52 - 91	25 - 137	74	62 - 87	35 - 142	0.838
Interleukin-6 (pg/mL)	0.91	0.81 - 1.29	0.28 - 4.96	1.38	1.08 - 1.82	0.39 - 7.17	1.36	0.96 - 2.10	0.84 - 3.28	0.048 ****

LNB^{-} = Lyme neuroborreliosis with less than four parallel persistent symptoms and/or neurological findings post treatment.

LNB^{+} = Lyme neuroborreliosis with more than four parallel persistent symptoms and/or neurological findings post treatment.

Q1-Q3 = Quartile one to three.

[a] Data missing on 2 controls and 1 LNB^{+} patient

[b] Data missing on 1 LNB^{+} patient

All p-values are without correction for age.

Significant group differences using Kruskal-Wallis test were further analysed using Mann-Whitney's U-test.

* Significantly lower age amongst healthy controls (p=0.001-0.023)

** Significantly higher in LNB^{+} compared to the other two groups (p=0.004-0.006 for heart rate and 0.002 for both differences for cortisol increment)

*** Significantly higher in LNB^{+} compared to healthy controls (p=0.003 for systolic blood pressure and 0.010 for follicle stimulating hormone)

**** Significantly higher in LNB^{-} compared to healthy controls (p=0.045)
